# Incremental Genetic Perturbations to MCM2-7 Expression and Subcellular Distribution Reveal Exquisite Sensitivity of Mice to DNA Replication Stress

**DOI:** 10.1371/journal.pgen.1001110

**Published:** 2010-09-09

**Authors:** Chen-Hua Chuang, Marsha D. Wallace, Christian Abratte, Teresa Southard, John C. Schimenti

**Affiliations:** Department of Biomedical Sciences and Center for Vertebrate Genomics, Cornell University, Ithaca, New York, United States of America; The Jackson Laboratory, United States of America

## Abstract

Mutations causing replication stress can lead to genomic instability (GIN). *In vitro* studies have shown that drastic depletion of the MCM2-7 DNA replication licensing factors, which form the replicative helicase, can cause GIN and cell proliferation defects that are exacerbated under conditions of replication stress. To explore the effects of incrementally attenuated replication licensing in whole animals, we generated and analyzed the phenotypes of mice that were hemizygous for *Mcm2*, *3*, *4*, *6*, and *7* null alleles, combinations thereof, and also in conjunction with the hypomorphic *Mcm4^Chaos3^* cancer susceptibility allele. *Mcm4^Chaos3/Chaos3^* embryonic fibroblasts have ∼40% reduction in all MCM proteins, coincident with reduced *Mcm2-7* mRNA. Further genetic reductions of *Mcm2*, *6*, or *7* in this background caused various phenotypes including synthetic lethality, growth retardation, decreased cellular proliferation, GIN, and early onset cancer. Remarkably, heterozygosity for *Mcm3* rescued many of these defects. Consistent with a role in MCM nuclear export possessed by the yeast Mcm3 ortholog, the phenotypic rescues correlated with increased chromatin-bound MCMs, and also higher levels of nuclear MCM2 during S phase. The genetic, molecular and phenotypic data demonstrate that relatively minor quantitative alterations of MCM expression, homeostasis or subcellular distribution can have diverse and serious consequences upon development and confer cancer susceptibility. The results support the notion that the normally high levels of MCMs in cells are needed not only for activating the basal set of replication origins, but also “backup” origins that are recruited in times of replication stress to ensure complete replication of the genome.

## Author Summary

Proper replication of the genome is essential for maintenance of the genetic material and normal cell proliferation. DNA replication can be compromised by exogenous factors and genetic disruptions. Such compromise can lead to disease such as cancer, which is characterized by genomic instability (an elevated mutation rate). Because the DNA replication apparatus is essential, relatively little is known about how genetic variants impact the health of whole animals. In this report, we studied mice bearing combinatorial mutations in a component of the replication apparatus, the MCM2-7 helicase. MCM2-7 is a complex of 6 proteins that are essential for initiating DNA replication along chromosomes, and to unwind the DNA during DNA replication. We find that although cells have excess amounts of MCM2-7 to support proliferation under normal circumstances, that incremental MCM depletions have multiple drastic effects upon the whole animal, including embryonic lethality, stem cells defects, and severe cancer susceptibility. Additionally, we report that mouse cells regulate and coordinate the levels of usable MCM proteins, both at the level of synthesis and also by regulating access to chromatin. The implication is that genetic variants that impact MCM levels, even to a minor degree, can translate into disease.

## Introduction

In late mitosis to early G1 phase of the cell cycle, DNA replication origins are selected and bound by the hexameric origin recognition complex (ORC; [1]). ORC then recruits the initiation factors CDC6 and CDT1, which are required for loading MCM2-7, thereby forming the “pre-replicative complex” (pre-RC). The formation of pre-RCs is termed origin “licensing” and this gives origins competency to initiate a single round of DNA synthesis before entering S phase. MCM2-7 is a hexamer of six distinct but structurally-related minichromosome maintenance (MCM) proteins (reviewed in [2]–[5]). *In vivo* and *in vitro* evidence indicates that the MCM2-7 complex is the replicative helicase [6]–[8].

MCM2-7 proteins are abundant in proliferating cells [9], and are bound to chromatin in amounts exceeding that which is present at active replication origins or required for complete DNA replication [10]–[14]. Although these and other studies showed that drastic decreases in MCMs are tolerated by dividing cells, there are certain deleterious consequences. In *Xenopus* extracts and mammalian cells, excess chromatin-bound MCM2-7 complexes occupy dormant or “backup” origins that are activated under conditions of replication stress, compensating for stalled or disrupted primary replication forks [11], [15]–[16]. The depletion of these backup licensed origins was associated with elevated chromosomal instability and susceptibility to replication stress, factors that might predispose to cancer.

In previous work, Shima *et al* found that a hypomorphic allele of mouse *Mcm4* (*Mcm4^Chaos3^*) caused high levels of GIN and extreme mammary cancer susceptibility in the C3HeB/FeJ background [17]. This provided the first concrete evidence that endogenous mutations in replication licensing machinery may have a causative role in cancer development. The ethylnitrosourea (ENU)-induced *Mcm4^Chaos3^* point mutation changed PHE to ILE at residue 345 (Phe345Ile). This amino acid is conserved across diverse eukaryotes and is important for interaction with other MCMs [18]. Budding yeast engineered to bear the orthologous mutation exhibit DNA replication defects and GIN [17], [19]. Surprisingly, MEFs from *Mcm4^Chaos3^* mice not only had reduced levels of MCM4, but also MCM7 [17], suggesting that the point mutation might destabilize the MCM2-7 complex. Subsequently, it was reported that mice containing 1/3 the normal level of MCM2 succumbed to lymphomas at a very young age, and had diverse stem cell proliferation defects [20]. These mice also had 27% reduced levels of MCM7 protein, and their cells exhibited decreased replication origin usage when under replication stress (treatment with hydroxyurea) conditions [21]. These studies imply that relatively modest decreases in any of the MCMs may be sufficient to cause cancer susceptibility, developmental defects, and GIN [20].

Here, we report that genetically-induced reductions of MCM levels in mice, achieved by breeding combinations of MCM2-7 alleles, caused several health-related defects including increased embryonic lethality, GIN, cancer susceptibility, growth retardation, defective cell proliferation, and hematopoiesis defects. Remarkably, genetic reduction of MCM3, which mediates nuclear export of excess MCM2-7 complexes in yeast [22], rescued many of these defects, presumably attributable to observed increases in chromatin-bound MCM levels. These data suggest that relatively minor misregulation or destabilization of MCM homeostasis can have serious consequences for health, viability and cancer susceptibility of animals.

## Results

### *Mcm4^Chaos3/Chaos3^* cells exhibit pan-reduction of total and chromatin-bound MCM2-7 due to decreased mRNA levels

**Figure 1:**
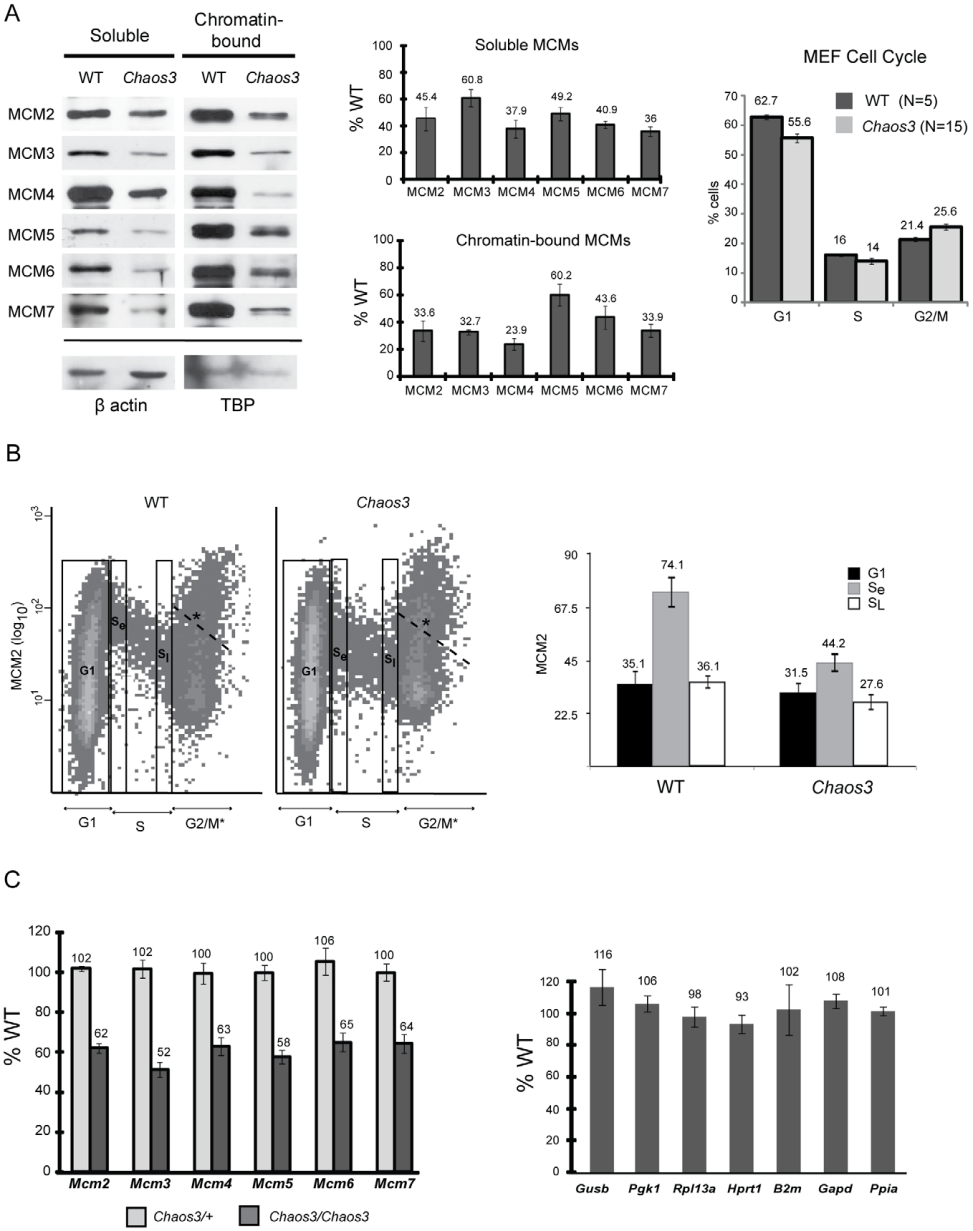
MCM2-7 proteins and mRNA are reduced in *Mcm4^Chaos3/Chaos3^* MEFs, particularly in early S phase. (A) Western blot analysis of MCM2-7 (left panel). Soluble or chromatin-bound protein was electrophoresced on PAGE gels, electrotransferred, and the blots were immunolabeled with the indicated antibodies. The bands correspond to the predicted molecular weights of these proteins, and for MCM2, the identity of the band was verifed by RNAi knockdown and transient overexpression in NIH 3T3 cells. TBP = TATA box binding protein. Quantification of Western blot data by densitometry is shown in the center panel. The amounts relative to WT cells (after normalization to the controls) are plotted. Error bars represent SEM, derived from 4 replicate experiments. The rightmost panel graphs the results of flow cytometric analysis of unsynchronized MEF culture cell cycle profiles, based on DNA content. (B) Flow cytometric quantification of MCM2 content (fluorescence intensity of antibody staining) is plotted on the Y axis, vs DNA content on the X axis. Plotted at the right is the mean fluorescent intensity of the 3 gated (boxed) regions from the flow data. S_e_ = early S phase; S_L_ = late S phase. The labeled cell cycle stages are based on DNA content. However, because light scatter was inadequate to distinguish individual nuclei from clumped nuclei or artifactual structures, the 4c category (denoted G2/M*) contains events other than G2/M nuclei. *We drew a dashed line representing an arbitrary cutoff above which contains such undefined events. (C) qRT-PCR analysis of Mcm mRNAs (left panel) and control genes (right panel), in the three indicated genotypes of MEFs. Relative transcript levels were normalized to β-actin. Charted are the percent levels of the indicated RNAs in mutant compared to WT (considered to be 100%). At least 3 replicate cultures were analyzed for each genotype. Error bars are SEM. In all panels, the raw data shown are from MEFs established from littermates. Furthermore, the replicates involved MEFs from pairs of littermates. Chaos3 = *Mcm4^Chaos3/Chaos3^*; WT = +/+.

To extend previous findings that *Mcm4^Chaos3Chaos3^* cells exhibited decreases in MCM4 and MCM7 protein, and to determine if the decreased levels were differentially compartmentalized in the cell, we quantified soluble and chromatin-bound MCM2-7 levels in mouse embryonic fibroblasts (MEFs) by Western blot analysis. As shown in Figure 1A, all MCMs were decreased in both compartments by at least 40% compared to WT cells. Because *Mcm4^Chaos3/Chaos3^* MEF cultures have slightly decreased proliferation and G2/M delay (Figure 1A and [17]), it is possible that the lower MCM levels in mutant MEFs are entirely attributable to growth defects. To test this, we assessed the levels of nuclear MCM2 in S-phase cells by flow cytometry (Figure 1B). Although MCM2 levels in WT and *Mcm4^Chaos3/Chaos3^* G1 nuclei were essentially the same (*P* = .65; t-test), mutant cells transitioned from G1 to S with 40% less nuclear MCM2 content than in WT (*P*<.02; t-test). The levels of nuclear MCM2 in WT decreased through S phase more sharply than in mutants, which transitioned to G2 with only ∼23% less than controls (Figure 1B). This differential decline is apparent in the flow plots, where WT cells exhibit a greater downward slope in the S compartment (Figure 1B). The decreases in MCM2 from early to late S were 51% in WT and 38% in mutants. The MCM2 intra-S modulation phenomenon is also addressed in subsequent experiments. The marked differences in nuclear MCM2 concentration between actively proliferating (S-phase) WT and mutant cells indicates that a biochemical or regulatory basis, rather than a population skewing, underlies the differences in protein levels.

Another possible explanation for the coordinated decrease in MCMs is that the mutant MCM4^Chaos3^ protein destabilizes the MCM2-7 hexamer and causes subsequent degradation of uncomplexed MCMs. Other groups reported that knockdown of *Mcm2*, *Mcm3*, *or Mcm5* in human cells decreased the amount of other chromatin-bound MCMs [15]–[16], leading to a similar proposition that the cause was MCM2-7 hexamer destabilization [16]. If true, then we would expect mRNA levels to be unchanged in mutant cells. To test this, we performed quantitative RT-PCR (qRT-PCR) analysis of *Mcm2-7*, and several control housekeeping genes in *Mcm4^Chaos3/Chaos3^* MEFs. Analysis of 5 littermate pairs of primary MEF cultures revealed that transcript levels for each of these genes in mutant cells was 51–65% of WT, similar to the protein decreases (Figure 1C). Levels of mRNA in the 7 housekeeping genes analyzed were not altered significantly (Figure 1C, right panel). This data suggest that either reduced MCM4 levels *per se*, or defects resulting from the *Mcm4^Chaos3^* allele, cause a decrease in the levels of all Mcm mRNAs. Interestingly, the mRNA reduction appears to occur post-transcriptionally, a phenomenon that is currently under investigation (Chuang and Schimenti, unpublished observations).

### Decreased Mcm gene dosages cause elevated chromosomal instability and *Mcm2*-specific pan-decreases in Mcm mRNAs

**Figure 2:**
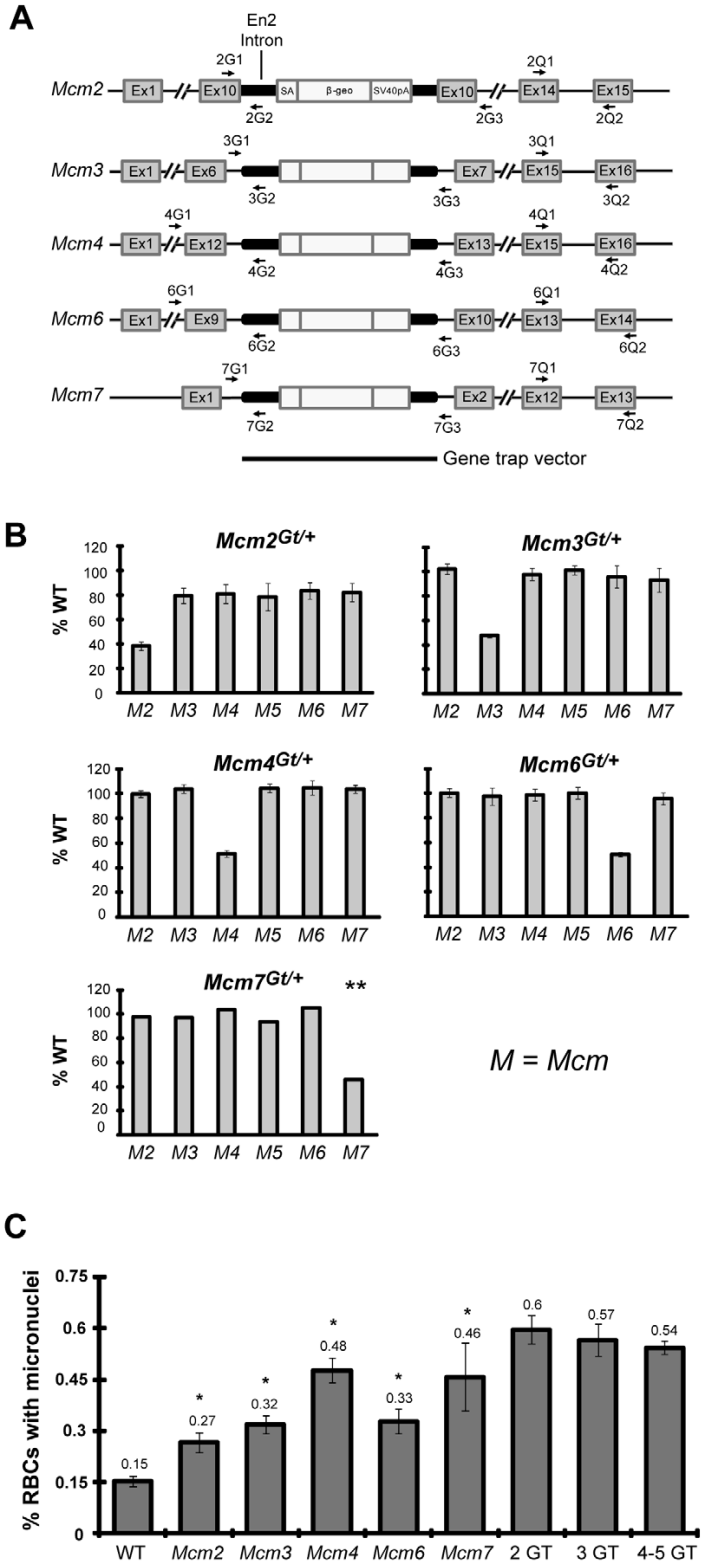
Mcm gene trap alleles and associated mRNA levels, peripheral blood micronuclei, and cancer frequency. (A) Genomic structures of mutated Mcm genes. Indicated is the intron/exon structure of each gene (not to scale), the locations of the gene trap insertions, and qPCR primer locations. (B) qRT-PCR analyses of MEF mRNA from gene trap heterozygotes. Charted are the percent levels of the indicated RNAs in mutant compared to WT (considered to be 100%). For all but *Mcm7*, the data were obtained from at least 3 MEF cultures from different embryos. The *Mcm7* data represents the average of three replicates from 1 MEF culture, hence there are no error bars. Otherwise, error bars show SEM. (C) Micronucleus levels in Mcm gene trap-bearing male mice. At least 5 animals were analyzed for each single gene trap mutant allele. The “2GT” (two gene trap) group contains: 4 mice doubly heterozygous for *Mcm2^Gt^* and *Mcm3^Gt^* (“Mcm2/3”), 4 Mcm2/4 mice, and 4 Mcm3/4 mice. The 3GT group contains: 4 Mcm2/3/4 mice, 1 Mcm2/3/6 mouse, 1 Mcm2/4/6 mouse, and 3 Mcm3/4/6 mice. The 4-5GT group contains: 3 Mcm2/3/4/6 mice, 1 Mcm2/3/6/7 mouse, and 2 Mcm2/3/4/6/7 mouse. SEM bars are shown.

The *Mcm4^Chaos3^* allele was identified in a forward genetic screen for mutations causing elevated micronuclei (MN) in red blood cells, an indicator of GIN [17]. While the altered MCM4^Chaos3^ protein may cause DNA replication errors as does a yeast allele engineered to contain the same amino acid change [19], it is also possible that the decrease in overall MCM levels in *Mcm4^Chaos3^* mutants contributes to, or is primarily responsible for, elevated S-phase DNA damage and GIN as is seen in various cell culture models (see Introduction). To test this possibility, we generated mice from ES cells bearing gene trap insertions in *Mcm2*, *Mcm3*, *Mcm6*, and *Mcm7* (Figure 2A; alleles are designated as *Mcm#^Gt^*). These gene traps are designed to disrupt gene expression by fusing the 5′ end of the endogenous mRNA (*via* use of a splice acceptor) to a vector-encoded reporter, resulting in a fusion protein lacking the C-terminal portion of the endogenous (MCM) protein. As with a previously-reported *Mcm4* gene trap [17], each of these alleles proved to be recessive embryonic lethal (Figure S1). Furthermore, each allele appeared to be a null, since mRNA levels in heterozygous MEF cultures were ∼50% lower than WT controls (Figure 2B). To determine if heterozygosity for various Mcms caused pan-decreases in Mcm mRNA levels as does homozygosity for *Mcm4^Chaos3^*, mRNA levels for each of the Mcm2-7 genes were also quantified. Whereas *Mcm2^Gt/+^* cells did show ∼20% decreases in the other Mcms, the *Mcm3*, *Mcm4*, *Mcm6* and *Mcm7* gene trap alleles did not (Figure 2B). Thus, it appears that the marked Mcm pan-decreases in *Mcm4^Chaos3/Chaos3^* cells are not due to decreased *Mcm4* RNA *per se*, but rather a response to replication defects cause by the mutant protein. Notably, the pan Mcm2-7 downregulation in *Mcm2^Gt/+^* cells is consistent with the observation that MCM7 is decreased in *Mcm2^IRES-CreERT2/IRES-CreERT2^* mice, although mRNA levels were not evaluated in that study [20].

After breeding the gene trap alleles into the C3HeB/FeJ genetic background for at least 2 generations (*Mcm4^Chaos3/Chaos3^* females get mammary tumors in this background), blood MN levels were measured. Heterozygosity for each allele caused an increase in the fraction of cells with MN (Figure 2C). Compound heterozygosity further increased MN on average, as did heterozygosity for 3 or more gene traps (Figure 2C), indicating that genetically-based decreases in any of the MCMs precipitate GIN.

### Genetic reductions of *Mcm2*, *Mcm6* or *Mcm7* in an *Mcm4^Chaos3/Chaos3^* background causes partial synthetic lethality, severe growth defects and (for *Mcm2*) dramatically accelerated cancer onset

**Figure 3:**
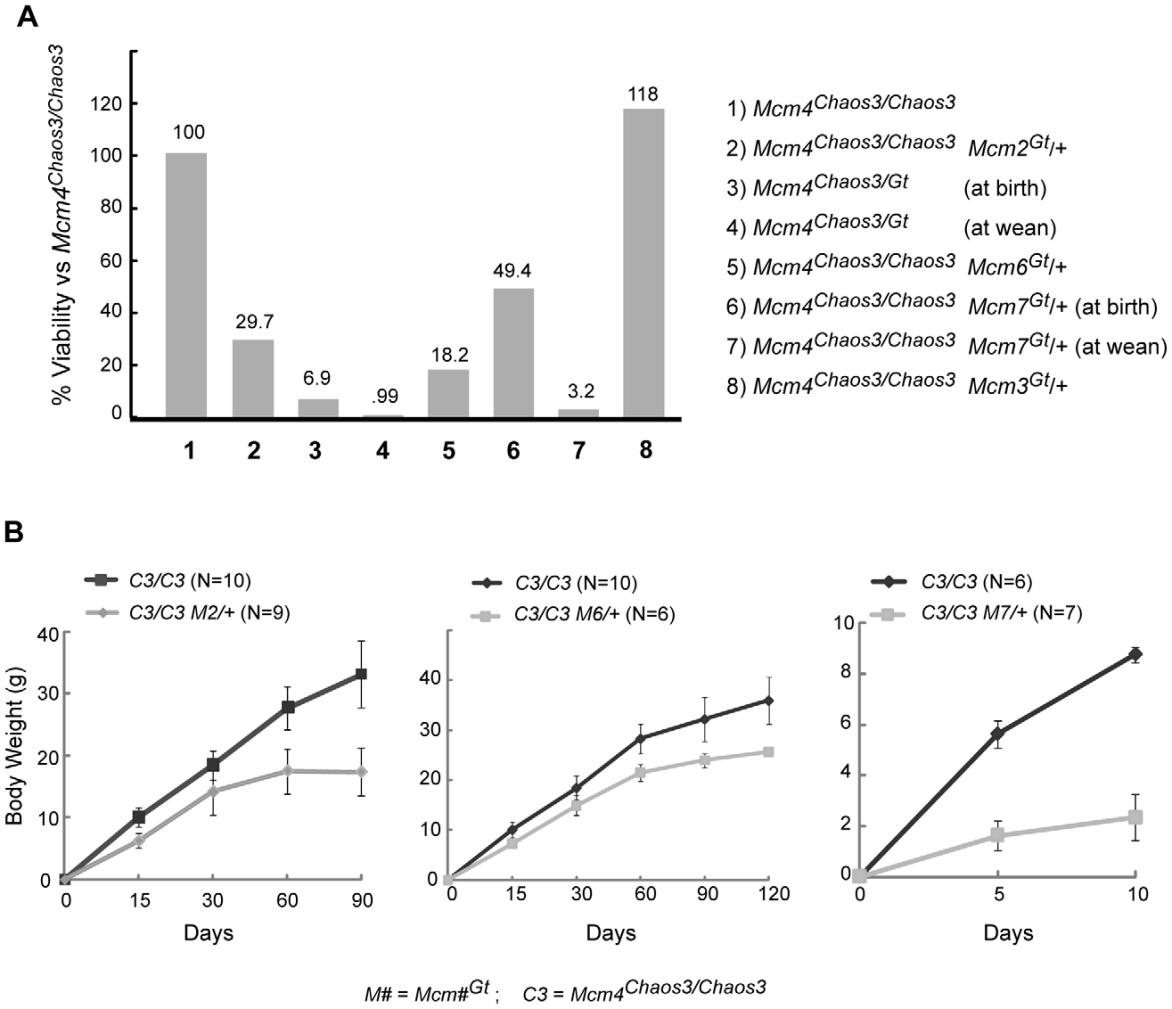
Synthetic lethality and growth retardation between *Mcm4^Chaos3^* and *Mcm2*, *Mcm6* and *Mcm7*. (A) Graphed are viability data from crosses presented in Figure S2, which includes statistics. Unless otherwise indicated, the values represent expected proportions of indicated genotypes that were present at wean. (B) Weights of surviving animals are graphed over time. SEM bars are shown.

As outlined above, previous studies showed that reductions of particular MCMs in cells or mice reduces the levels of other MCMs, causing GIN, cancer, and developmental defects. However, the reduction in MCM levels required to precipitate these consequences, and whether there is a threshold effect, is unclear. To explore the consequences of incremental MCM reductions on viability and cancer in mice, we crossed the *Mcm4^Chaos3^* and gene trap alleles into the same genome. In the case of *Mcm2*, there was a striking and highly significant shortfall of *Mcm4^Chaos3/Chaos3^ Mcm2^Gt/+^* offspring at birth (Figure 3A; Figure S2). Heterozygosity for *Mcm2^Gt^* itself was not haploinsufficient, as indicated by Mendelian transmission of *Mcm2^Gt^* in crosses of heterozygotes to WT (119/250; χ^2^ = 0.448). These results demonstrate that there is a synthetic lethal interaction between *Mcm4^Chaos3^* and *Mcm2^Gt^* that is related to MCM2 levels. Additionally, the surviving *Mcm4^Chaos3/Chaos3^ Mcm2^Gt/+^* offspring were severely growth retarded; males weighed ∼50% less than *Mcm4^Chaos3/Chaos3^* siblings (Figure 3B; this genotype causes disproportionate female lethality). Another indication of a quantitative MCM threshold effect is that C3H-*Mcm4^Chaos3/Chaos3^* mice are developmentally normal, but *Mcm4^Chaos3/Gt^* animals die *in utero* or neonatally (Figure 3A) [23].

The synthetic interaction between *Mcm4^Chaos3^* and *Mcm2^Gt^* might be specific, or it may reflect a general consequence of reduced replication licensing (and consequent elevated replication stress). We therefore tested whether hemizygosity for *Mcm3*, *Mcm6* or *Mcm7* would also cause synthetic phenotypes in the *Mcm4^Chaos3/Chaos3^* background. The *Mcm4^Chaos3/Chaos3^ Mcm6^Gt/+^* genotype caused highly penetrant embryonic lethality; only 10% of the expected number of such animals survived to birth (Figure 3A; Figure S2). The *Mcm4^Chaos3/Chaos3^ Mcm7^Gt/+^* genotype caused both embryonic and postnatal lethality. The number of liveborns was ∼50% of the expected value, and only 8% of those (5/62) survived to weaning (Figure 3A; Figure S2). Additionally, as with *Mcm2*, hemizygosity for *Mcm6^Gt^ and Mcm7^Gt^* in the *Mcm4^Chaos3/Chaos3^* background caused growth retardation (Figure 3B). The decrease in male weight was ∼20% and ∼80% respectively, compared to *Mcm4^Chaos3/Chaos3^* siblings at the oldest age measured (*Mcm4^Chaos3/Chaos3^ Mcm7^Gt/+^* animals died before wean, so the oldest weights were taken at 10 dpp). In contrast to the synthetic phenotypes with *Mcm2*, *4*, *6* and *7*, there was no significant decrease in viability (Figure 3A) or weight (not shown) in *Mcm4^Chaos3/Chaos3^ Mcm3^Gt/+^* mice. This seeming inconsistency is addressed in the following section.

**Figure 4:**
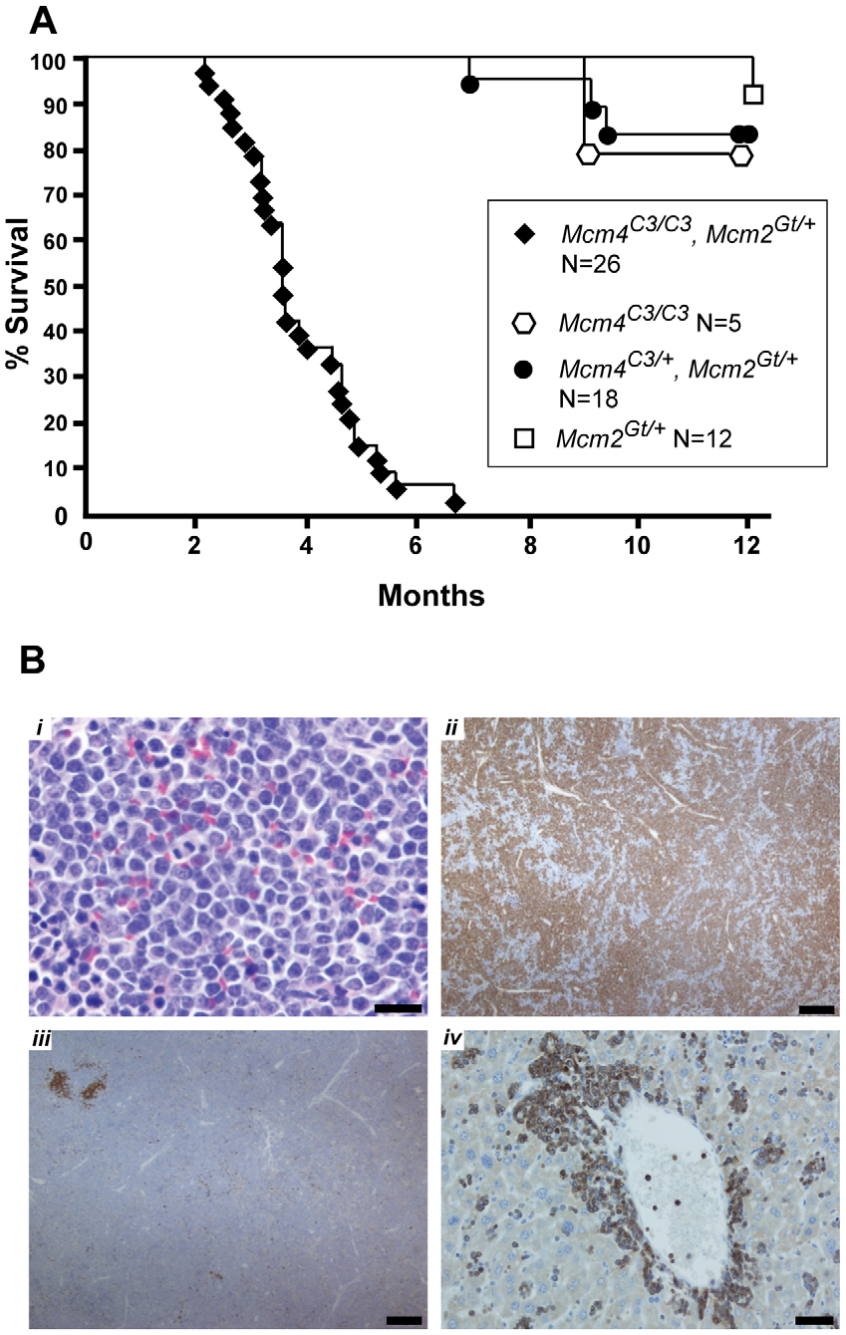
Premature morbidity and cancer susceptibility in *Mcm4^Chaos3/Chaos3^ Mcm2^Gt/+^* mice. (A) Kaplan-Meier survival plot of the indicated genotypes. Animals of both sexes are combined. “C3” = *Chaos3*. (B) Spleen and liver histopathology of a *Mcm4^C3/C3^ Mcm2^Gt/+^* male diagnosed with T cell leukemic lymphoma. ***i*** *.* H&E stained spleen. Neoplastic cells have abundant cytoplasm, 1–2 nucleoli and a high mitotic rate, consistent with lymphoblastic lymphoma. Bar = 20 µm. ***ii*** *.* Neoplastic cells in spleen demonstrate immunoreactivity with anti-CD3 (brown; immunoperoxidase staining with DAB chromogen & hematoxalin counterstain), indicating T lymphocytes. Bar = 200µm. ***iii*** *.* In spleen, immunoreactivity (brown) with anti-PAX-5 (a B cell marker) is limited to follicular remnants and scattered individual cells. Bar = 200 µm. ***iv*** *.* In liver, neoplastic cells surround central veins and expand sinusoids (see also Figure S4) and demonstrate immunoreactivity (brown) with the anti-CD3 T lymphocyte marker. Bar = 50 µm.

As mentioned earlier, mice with ∼35% of WT MCM2 protein, but not 62%, showed early latency (10–12 week) lymphoma susceptibility [20]. To identify if there is a critical MCM threshold for cancer susceptibility, we aged a cohort of *Mcm2^Gt/+^* mice, representing approximately intermediate MCM2 levels. As shown in Figure 4A, these animals did not show a dramatic cancer-related mortality in the first 12 months of life. However, we did find that ∼3/4 of these animals had tumors at death or necropsy by 18 months of age (data not shown). These combined data are suggestive of a potential gradient of susceptibility, but that there is a critical minimum threshold of MCM levels, between ∼35 and 50% in the case of MCM2, required to avoid early cancer and other developmental defects.

To further resolve this phenomenon, surviving *Mcm4^Chaos3/Chaos3^ Mcm2^Gt/+^* mice were aged and monitored. They began dying at 2 months of age, and all were dead (or sacrificed when they appeared moribund) by 7 months (Figure 4A). Gross necropsy and histopathological analyses revealed or suggested lymphomas/leukemias in 20 of these animals (summarized in Table S1 with histological examples in Figure S3; detailed histopathology analysis of a T cell leukemic lymphoma is presented in Figure 4B). Six of these had chest tumors that were likely thymic lymphomas. The cause of death for the remaining 7 animals was undetermined. Consistent with previous studies [17], most *Mcm4^Chaos3/Chaos3^* mice hadn't yet succumbed from tumors or other causes by 12 months of age. Additional animals of these genotypes are incorporated in Figure 6, but histopathological analyses weren't conducted. These data show clearly that removing a half dose of MCM2 from *Mcm4^Chaos3/Chaos3^* cells is sufficient to produce greatly elevated cancer predisposition to the already-underrepresented survivors at wean. *Mcm4^Chaos3/Chaos3^ Mcm2^Gt/+^* MEFs had 45% the amount of *Mcm2* mRNA as *Mcm4^Chaos3/Chaos3^* cells (Figure 7C), which already had a 38% reduction compared to WT (Figure 1). Thus, *Mcm2* RNA was reduced to ∼17% of WT. To determine if elevated GIN might be responsible for the cancer susceptibility phenotype, we measured erythrocyte MN. Whereas the percentage of micronucleated RBCs in *Mcm4^Chaos3/Chaos3^* mice was 4.18±0.26 (mean±SEM, N = 12), *Mcm4^Chaos3/Chaos3^ Mcm2^Gt/+^* mice averaged 5.85±0.47 (N = 16), indicating a synergistic increase (*P*<0.01). Overall, the data support the notion that in whole animals, reduction of MCMs to under 50% of WT causes severe developmental and physiological problems.

### Rescue of phenotypic defects in *Mcm4^Chaos3/Chaos3^* and *Mcm4^Chaos3/Chaos3^ Mcm2^Gt/+^* mice by reducing *Mcm3* genetic dosage

The data reported here and elsewhere [17], [20] support a model where phenotypic severity is proportionally related to MCM concentrations. However, our genetic experiments uncovered one notable exception: hemizygosity for *Mcm3* did not cause any severe haploinsufficiency phenotypes (increased lethality and decreased weight) as did *Mcm2/6/7* in the *Mcm4^Chaos3/Chaos3^* background, or *Mcm4^Gt^* in *trans* to *Mcm4^Chaos3^* (Figure 3A; Figure S2). Since extreme reductions of MCM3 in cultured human cells caused GIN and cell cycle arrest [16], the absence of synthetic effects with *Mcm^Chaos3^* led us to hypothesize that either mice are more tolerant to lower levels of this particular MCM, or that MCM3 is present in a stoichiometric excess compared to the other MCMs, at least in a subset of cell types. To explore these issues we performed additional phenotype analyses, and also sought to uncover potential effects of MCM3 reduction by reducing other MCMs simultaneously.

**Figure 5:**
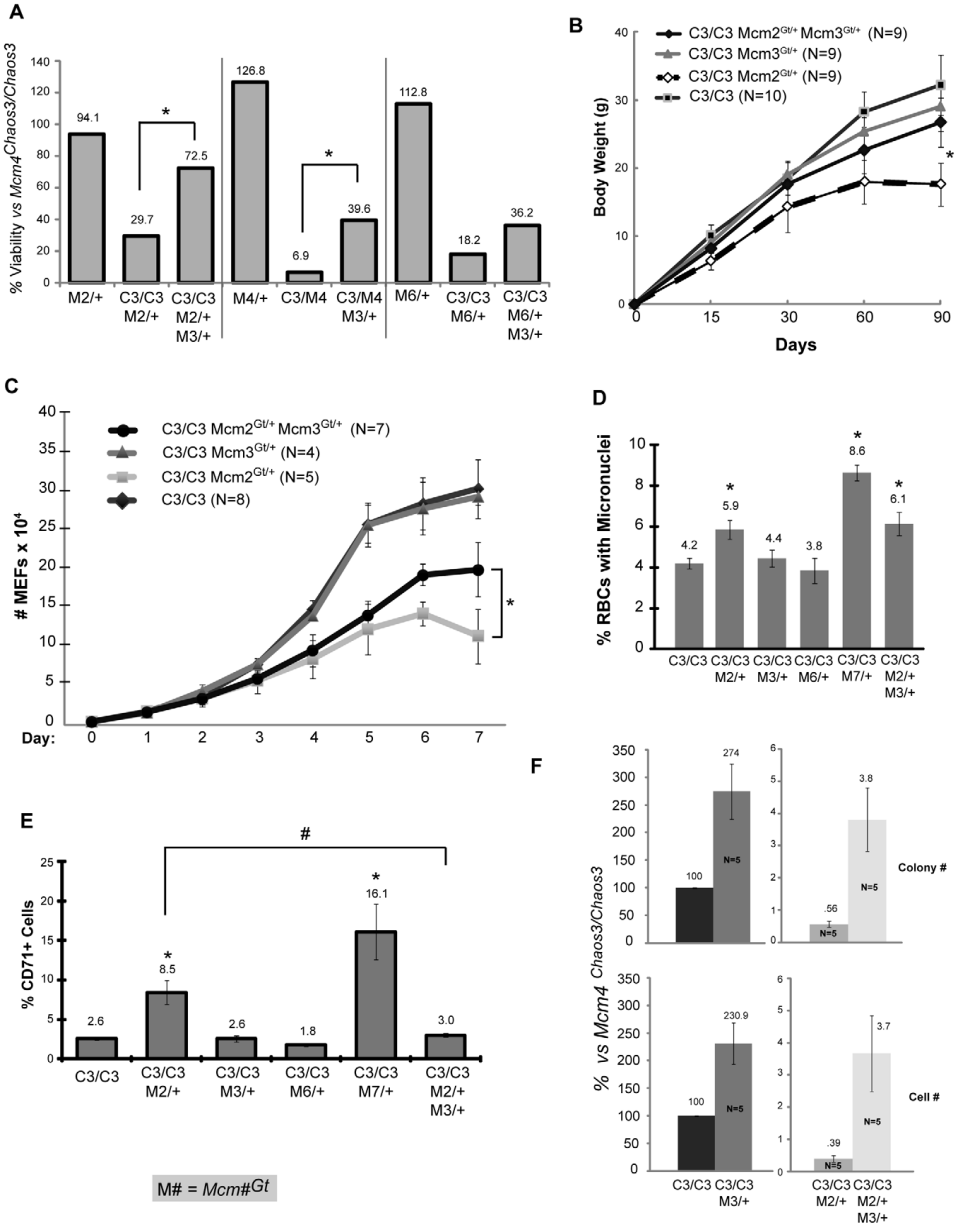
Rescue of phenotypes by *Mcm3* hemizygosity. (A) Heterozygosity for *Mcm3^Gt^* rescues the low viability of various mutant genotypes (asterisk indicates significance at *P*<0.05 by FET). The raw data are presented in Figure S4. (B) Male body weights of combination mutant mice. The weights of *Mcm4^Chaos3/Chaos3^ Mcm2^Gt/+^ Mcm3^Gt/+^* mice are significantly higher (asterisk; *P*<0.01, Student's t-test) at 90 days than *Mcm4^Chaos3/Chaos3^ Mcm2^Gt/+^* mice. Error bars represent SEM. (C) *Mcm4^Chaos3/Chaos3^ Mcm2^Gt/+^* MEF proliferation defects are partially rescued by *Mcm3* hemizygosity. The effect is significant after 6 days in culture (*P*<0.05, Student's t-test; Error bars represent SEM). (D) Micronucleus levels in *Mcm4^Chaos3/Chaos3^* mice bearing additional gene trap alleles. At least 5 males were analyzed for each genotype. Error bars represent SEM. Asterisk indicates *P*<0.05 (student's t-test.) compared to *Mcm4^Chaos3/Chaos3^* alone. (E) CD71+ reticulocyte ratios in mutant male mice. At least 5 animals were analyzed from each class. The samples are identical to those in “D”. All scored cells were anucleate peripheral blood cells Error bars represent SEM. Asterisks and “#” indicate *P*<0.05 (Student's t-test) when compared to *Mcm4^Chaos3/Chaos3^* and *Mcm4^Chaos3/Chaos3^ Mcm2^Gt/+^* cohorts, respectively. (F) *Mcm2* hemizygosity decreases efficiency of reprogramming *Mcm4^Chaos3/Chaos3^* MEFs into iPS cells, and *Mcm3* hemizygosity significantly increases reprogramming efficiency. Two methods of quanitifying reprogramming were used as described in Materials and Methods. “Cell number” refers flow cytometric quantification of LIN28/SSEA1 double positive cells from primary cultures of reprogrammed MEFs. Relative reprogramming efficiencies were normalized to *Mcm4^Chaos3/Chaos3^* MEFs (considered to be 100%). Error bars represent SEM. All samples within quantification class are significantly different from one another (*P*<0.05, Student's t-test). C3 = *Mcm4^Chaos3^*; M = *Mcm*.

Strikingly, rather than exacerbating the synthetic lethality in *Mcm4^Chaos3/Chaos3^ Mcm2^Gt/+^* mice, *Mcm3^Gt^* heterozygosity significantly *rescued* their viability to 72.5% from 29.7% (Figure 5A and Fig S3). Not only was viability rescued, but also growth (weight) of *Mcm4^Chaos3/Chaos3^ Mcm2^Gt/+^ Mcm3^Gt/+^* survivors compared to *Mcm4^Chaos3/Chaos3^ Mcm2^Gt/+^* animals produced from the same matings (Figure 5B). *Mcm3* hemizygosity also significantly rescued the near 100% lethality of *Mcm4^Chaos3/Gt^* animals (nearly 6 fold increased viability), and doubled the viability of *Mcm4^Chaos3/Chaos3^ Mcm6^Gt/+^* mice (Figure 5A; Figure S3). Rescue of *Mcm4^Chaos3/Chaos3^ Mcm7^Gt/+^* was not observed (not shown).

The rescue of the reduced growth phenotype by *Mcm3* hemizygosity led us to evaluate the proliferation of compound mutant cells. Whereas *Mcm4^Chaos3/Chaos3^* and *Mcm4^Chaos3/Chaos3^ Mcm3^Gt/+^* primary MEFs proliferated at identical rates, *Mcm4^Chaos3/Chaos3^ Mcm2^Gt/+^* MEFs showed a severe growth defect beginning ∼5 days in culture (Figure 5C). As with whole animals, MEF growth was partially but significantly rescued by *Mcm3* hemizygosity.

Since the *Mcm4^Chaos3^* and *Mcm2^Gt^* alleles causes elevated GIN (micronuclei in RBCs), we considered the possibility that the *Mcm3* rescue effect might be related to an attentuation of GIN. Accordingly, we measured MN levels in *Mcm4^Chaos3/Chaos3^* mice with different combinations of other Mcm mutations. As shown in Figure 5D, hemizygosity for *Mcm2* and *Mcm7* caused a significant elevation in MN levels, unlike *Mcm3*. However, the increased MN in *Mcm4^Chaos3/Chaos3^ Mcm2^Gt/+^* was not rescued by *Mcm3* hemizygosity. This suggests that the synthetic lethality and mouse/cell growth defects are not related to GIN *per se*. However, in the course of measuring MN in enucleated peripheral blood cells, we noticed that the ratio of CD71+ cells was significantly higher in both *Mcm4^Chaos3/Chaos3^ Mcm2^Gt/+^* and *Mcm4^Chaos3/Chaos3^ Mcm7^Gt/+^* mice (3.3 and 6.2 fold, respectively; Figure 5E). This increase in the ratio of reticulocytes (erythrocyte precursors; immature RBCs) to total RBCs is characteristic of anemia. Hemizygosity for *Mcm3*, which alone had no effect on CD71 ratios of Chaos3 mice, corrected completely this abnormal phenotype in *Mcm4^Chaos3/Chaos3^ Mcm2^Gt/+^*animals (Figure 5E).

Because MCM2-depleted mice were reported to have stem cell defects [20], and *Mcm4^Chaos3/Chaos3^ Mcm#^Gt/+^* mice had clear developmental abnormalities, we examined the efficiency of reprogramming mutant MEFs into induced pluripotent stem cells (iPS). The efficiency was quantified using either : 1) iPS-like colony formation, or 2) cells counts of SSEA1 and LIN28 positive cells by flow cytometry. Both gave similar results. *Mcm4^Chaos3/Chaos3^ Mcm2^Gt/+^* cells were severely compromised in the ability to form iPS cells compared to *Mcm4^Chaos3/Chaos3^* (∼200 fold less efficient; Figure 5F). However, additionally reducing *Mcm3* by 50% increased iPS formation from both *Mcm4^Chaos3/Chaos3^*and *Mcm4^Chaos3/Chaos3^ Mcm2^Gt/+^* MEFs by ∼2.5 and 10 fold, respectively.

**Figure 6:**
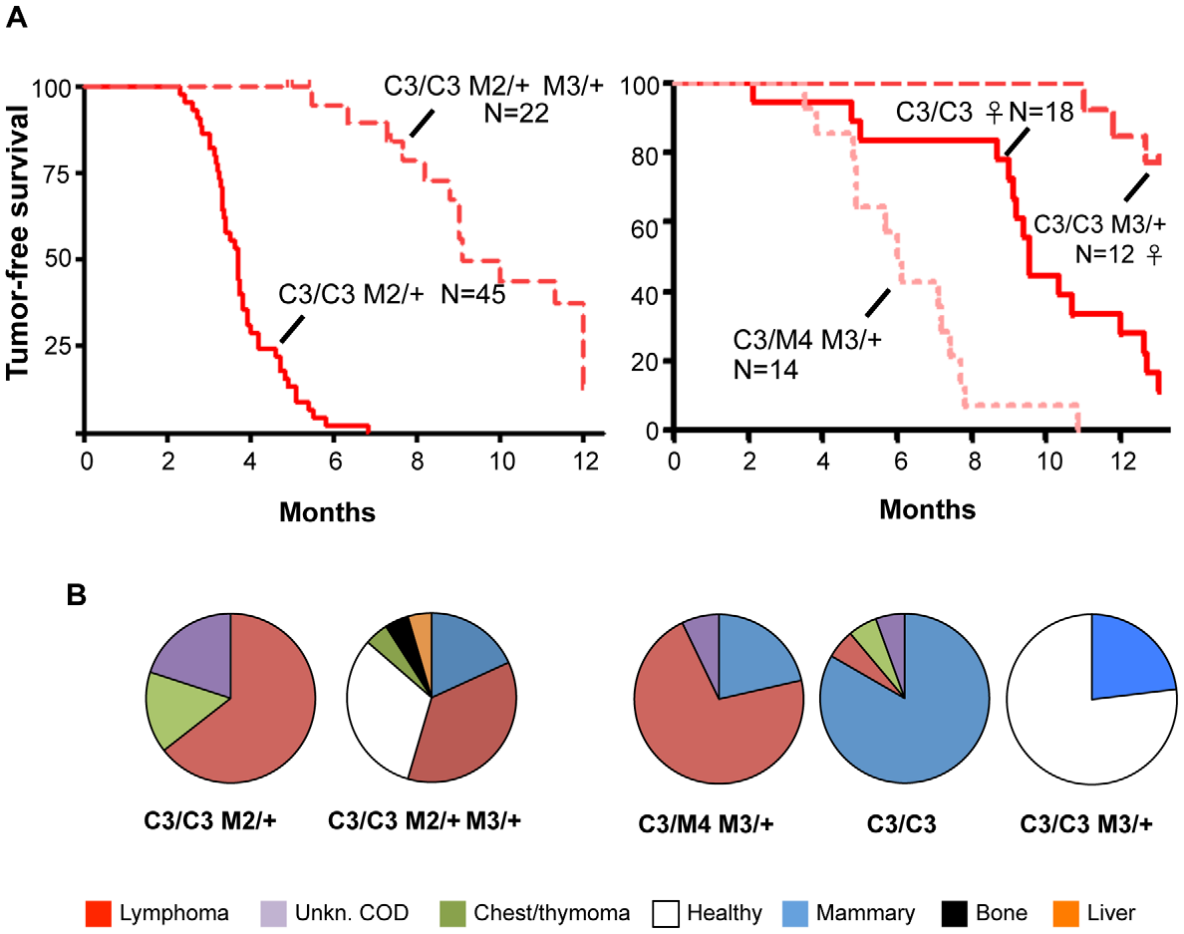
Inhibition of Chaos3 cancers by MCM3 reduction. (A) Kaplan-Meier graphs of cohorts of the indicated genotypes. C3 = *Mcm4^Chaos3^*; M# = *Mcm#^Gt^*. In the left panel, the experiment was terminated at 12 months, with ∼1/3 animals tumor-free and healthy at the time (see “B”). Unless otherwise indicated, the cohorts contained both sexes. (B) Pie charts of cancer types in mice from “A.” COD = cause of death; Unkn = unknown. Classification of cancer types was assigned during necropsy, not from histological analysis.

Finally, we found that reduced MCM3 levels could rescue the cancer susceptibility of two different Chaos3 models. As shown earlier (Figure 4), *Mcm4^Chaos3/Chaos3^ Mcm2^Gt/+^* mice were highly cancer-prone with an average latency of <4 months. When a dose of Mcm3 was removed from mice of this genotype, lifespan was extended dramatically in both sexes as a consequence of delayed cancer onset, and the cancer spectrum shifted from lymphoma/thymoma towards mammary tumors (Figure 6A). Additionally, hemizygosity of *Mcm3* delayed (or eliminated) the onset of mammary tumorigenesis in *Mcm4^Chaos3/Chaos3^* females by ∼4 or more months (Figure 6B). However, although *Mcm3* hemizygosity rescued viability of *Mcm4^Chaos3/Gt^* mice (Figure 5A), these animals were cancer prone with a shorter latency (by ∼6 months) and different spectrum (primarily lymphomas) than *Mcm4^Chaos3^* homozygotes.

### Decreased MCM3 increases chromatin-bound levels of other MCMs

**Figure 7:**
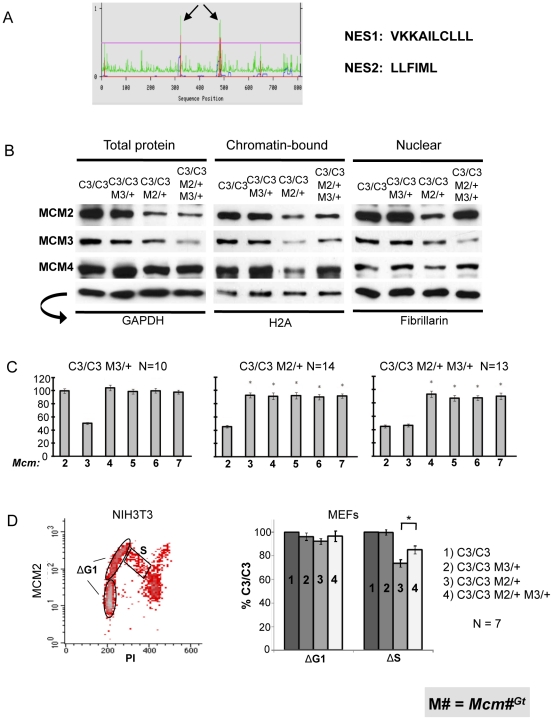
MCM3 regulates nuclear and chromatin-bound MCM levels. (A) Predicted nuclear export sequences (NES) in mouse MCM3 (see text). (B) Western blot analysis of MCM2/3/4 in the indicated genotypes of MEFs. Three different protein fractions were examined, with the indicated (arrow) loading controls at the bottom. (C) qRT-PCR analysis of Mcm2-7 mRNAs in MEFs of the indicated genotypes. (D) Nuclear MCM2 dynamics during the cell cycle. The flow plot is of isolated nuclei stained for DNA content (PI = propidium iodide) on the X-axis, and MCM2 on the Y-axis. NIH3T3 cells show dramatically the decrease in nuclear MCM2 through S phase. Flow cytometric data from the 4 MEF genotypes indicated in the right panel were used to calculate two values, ΔG1 and ΔS. The regions for the calculation of these values are indicated, and the values plotted in the right panel. The G1 (1N DNA content) phase nuclei were divided into two equal groups based on MCM2 signal intensity (Y-axis): the lower half, considered to be early-G1, and the upper half, considered to be late-G1. The ΔG1 value was calculated as the difference between the early and late MCM2 signal intensity averages. The ΔS value was calculated as : (average MCM2 intensity in the S population) – (early G1 average intensity). C3 = *Mcm4^Chaos3^*; M = *Mcm*. C3 is set at 100%. The asterisk indicates significance by Student's t-test (*P*<0.05).

We considered two possibilities to explain the surprising phenotypic rescues of reduced MCM genotypes (*Mcm4^Chaos3/Chaos3^* ; *Mcm4^Chaos3/Chaos3^* Mcm2/6^Gt/+^ ; *Mcm4^Chaos3/Gt^*) by additional MCM3 reduction (*Mcm3^Gt/+^*). One is that the phenotypes are related to altered stoichiometry of MCM monomers, and that disproportionally high amounts of MCM3 relative to MCM4 and MCM2/6/7 have a dominant negative effect. However, as demonstrated above, levels of MCM3 are proportionally reduced in *Mcm4^Chaos3/Chaos3^* cells (Figure 1). The second possibility is that decreased levels of MCM3 leads to a favorable change in the amounts or subcellular localization of MCMs. Various experiments have indicated that MCM2-7 hexamers or subcomplexes must be assembled in the cytoplasm before nuclear import in yeast [4], and in mice, nuclear import appears to require MCM2 and MCM3 [24]. MCMs shuttle between the nucleus and cytoplasm during the cell cycle in *S. cerevisiae*. Although in most other organisms MCMs are reported to be predominantly and constitutively nuclear localized throughout the cell cycle, dynamic redistribution between the nucleus and cytoplasm has been observed in hormonally-treated mouse uterine cells [25]. In budding yeast, nuclear export is dependent upon Mcm3, which has a nuclear export signal (NES) that is recognized by Cdc28 to promote export of MCM2-7 [22]. Analysis of mouse and human MCM3 using NES prediction software (www.cbs.dtu.dk/services/NetNES/) [26] revealed the presence of homologously-positioned, leucine-rich potential NESs (Figure 7A). Therefore, we hypothesized that the rescue of phenotypes by *Mcm3* hemizygosity is due to decreased MCM protein export from the nucleus, or alternatively, increased nuclear import or stabilization that allows greater access of all MCMs for licensing chromatin.

To explore this hypothesis, we performed Western blot analysis of MCM levels in *Mcm4^Chaos3/Chaos3^* MEFs with or without the *Mcm3^Gt^* and/or *Mcm2^Gt^* alleles, and examined the effects of *Mcm3* dosage on the levels of nuclear and chromatin-bound MCM2 and MCM4. The results are presented in Figure 7B. In all cases, the genetic reductions of *Mcm2* and *Mcm3* led to corresponding decreases in the cognate mRNA levels (Figure 7C), with only minor additional decreases of other MCM mRNAs (beyond that already caused by homozygosity for *Mcm4^Chaos3^*) occuring in the context of *Mcm2* hemizygosity (similar to *Mcm2^Gt/+^* MEFs in Figure 2B). The overall levels of total, nuclear, and chromatin-bound MCM2 and MCM4 were unaffected by hemizygosity of *Mcm3* in *Mcm4^Chaos3/Chaos3^* cells (Figure 7B). When *Mcm2* levels were genetically reduced by half, a condition causing the severe phenotypic effects described earlier, this caused a marked decrease in the level of chromatin-bound MCM3 and MCM4 (in addition to MCM2 itself), although total and nuclear MCM3/4 levels were affected to a lower degree or not at all. Strikingly, the decreased levels of chromatin-bound MCM2/3/4 in *Mcm4^Chaos3/Chaos3^ Mcm2^Gt/+^* MEFs were reversed by *Mcm3* heterozygosity, but levels of total MCM2 and MCM4 were not restored. The increase of chromatin-bound MCMs occured despite the presence of less MCM3, suggesting that MCM3 is present at levels in excess of that needed to bind chromatin, presumably for pre-RC formation in the context of the MCM2-7 hexamer. In conclusion, a 50% reduction in total MCM3 increases MCM2/4 loading onto chromatin when MCM2 is otherwise limiting, and this rescue is associated with amelioration of several phenotypes.

We found that elevation of nuclear MCMs in the *Mcm3^Gt/+^* MEFs was often (as shown in Figure 7B), but not consistently elevated across samples by Western analysis (not shown). Therefore, we quantified MCM2 during the cell cycle by flow cytometric analysis of nuclei from 7 replicate MEF cultures. Similar to WT MEFs (examples in Figure 1B), NIH3T3 cells showed a decrease of nuclear MCM2 during S phase progression (Figure 7D, left panel). However, all genotypes with in the *Mcm4^Chaos3/Chaos3^* background had a reduced decline. Thus, for comparative quantitation across genotypes, we compared the levels of MCM2 levels at the beginning of G1 *vs.* that in S phase (regions used for these calculations are indicated in the left panel), using the calculation described in the Figure 7 legend. The data are graphed in the right panel. The data revealed that regardless of genotype, the difference in average amounts of nuclear MCM2 at the beginning and end of G1 (ΔG1) did not vary. Compared to *Mcm4^Chaos3/Chaos3^*, cells lacking 1 dose of *Mcm2* had relatively lower levels of S phase MCM2 (ΔS) compared to early G1. Additional removal of an *Mcm3* dose partially rescued the ΔS value, indicating that these cells had ∼16% more nuclear MCM2 in S phase compared to *Mcm4^Chaos3/Chaos3^* cells hemizygous for *Mcm2* alone, despite overall reduced MCM2 levels in the cell (Figure 7B, left panel).

## Discussion

MCM2-7 proteins exist abundantly in proliferating cells and are bound to chromatin in amounts exceeding that required to license all replication origins that initiate DNA synthesis [9]–[12], [14]. The role of excess chromatin-bound MCM2-7 has been a mystery referred to as the “MCM paradox” [27], perpetuated by observations that drastic MCM reductions in certain systems can be compatible with normal DNA replication or cell proliferation [13], [28]–[30]. However, these circumstances are not universal, and reductions are not entirely without consequences. Early studies showed that a reduction in MCMs resulted in decreased usage of certain ARSs [12] and conferred genome instability [31] in yeasts. In cell culture systems, depletion of certain MCMs have been found to cause cell cycle defects, checkpoint abberations and GIN [13], [16]–[17], [29], [32].

Recent work has shed light on aspects of the MCM paradox. Using *Xenopus* egg extracts attenuated for licensing by addition of geminin (an inhibitor of CDT1, which is required for MCM loading onto origins), one study proposed that excess chromatin-bound MCM2-7 complexes license “dormant” origins that can be activated to rescue stalled or damaged replication forks, a situation that can become important under conditions of replication stress [11]. Similar results were subsequently reported for human cells depleted of MCMs by siRNA [15]–[16], and for replication stressed MCM2-deficient MEFs [21]. Our finding that nuclear MCM2 levels decrease as S-phase progresses, and moreso in WT than in *Mcm4^Chaos3/Chaos3^* MEFs, is consistent with the dormant origin hypothesis. The decrease may reflect displacement of dormant hexamers by active replisomes, followed by subsequent degradation or nuclear export. If WT nuclei have more dormant licensed origins than Chaos3 mutants, then WT cells would be expected show a greater loss of MCMs.

The isolation of *Mcm4^Chaos3^* provided the first demonstration that mutant alleles of essential replication licensing proteins can cause GIN and cancer [17]. Diploid budding yeast containing the same amino acid change in scMcm4 as the mouse *Mcm4^Chaos3^* exhibited Rad9-dependent G2/M delay (Rad9 is a DNA damage checkpoint protein), elevated mitotic recombination, chromosome rearrangements, and intralocus mutations [19] (Li, X. and Tye, B., personal communication). One explanation for these outcomes is that the *Chaos3* mutation impairs MCM4 biochemically in a manner leading to elevated replication fork defects, and that these defects lead to the GIN and cancer phenotypes. Alternatively, and/or in addition, the observed associated pan-reductions of MCMs in mouse cells [17] raised the possibility that decreased replication licensing might be the primary or ancillary cause for the mouse phenotypes.

The subsequent finding that mice (*Mcm2^IRES-CreERT^*) containing ∼1/3 the normal level of MCM2 had GIN and and cancer lent support for the idea that reductions in MCMs contribute to the Chaos3 phenotypes [20]. Although amounts of all MCMs were not investigated in *Mcm2^IRES-CreERT/IRES-CreERT^* mice, 65% reduction of MCM2 caused a reduction of dormant replication origins in MEFs that were replication stressed by hydroxyurea [21]. In *Mcm4^Chaos3/Chaos3^* mice, we hypothesize that in the context of *Mcm2*, *6* or *7* heterozygosity, which further reduces overall and chromatin-bound MCM levels below that already caused by *Mcm4^Chaos3^* (measured to be <20% of WT mRNA levels for *Mcm2*), MCMs are reduced to a degree that compromises cell proliferation. This then translates into the various developmental defects and increased cancer susceptibility we observed. Whatever the exact mechanistic cause of these phenotypes, it is clear that the phenotypes are related to reduction of one or more MCMs below a threshold level that is <50%. The severe developmental consequences of MCM depletion in mice suggests that certain cell types in the developing embryo are highly sensitive to the effects of replicative stress, and/or that relatively minor cell growth perturbations of such cells are not well-tolerated in the context of complex, rapidly-occuring developmental events. The molecular basis for these phenotypes does not appear to be directly related to GIN, because whereas *Mcm3* hemizygosity rescued several phenotypes, and delayed cancer latency in *Mcm4^Chaos3/Chaos3^* mice, it did not concommitantly decrease MN. This suggests that phenotypes such as decreased proliferation and embryonic death are caused by genetically-induced replication stress, moreso (or in addition to) than GIN alone.

Our genetic studies indicate that there is a quantitative MCM threshold required for embryonic viability, as demonstrated by the synthetic lethalities we observed when combining homozygosity of *Mcm4^Chaos3^* with *Mcm2^Gt^*, *Mcm6^Gt^ or Mcm7^Gt^* heterozygosity, but not in the heterozygous single mutants. Additionally, the *Mcm4^Chaos3/Gt^* genotype, which reduced MCM levels below 50%, caused embryonic and neonatal lethality [17]. Underscoring the exquisite sensitivity of whole animals to subtle perturbations in the DNA replication machinery were the remarkable phenotypic rescues (viability, growth, iPS efficiency, etc.) by *Mcm3* hemizygosity. The decreased MCM dosage led to increases in S phase nuclear MCMs and chromatin-bound MCMs, presumably reflecting increased replication origin formation. The various single and compound mutants described here and elsewhere [20], which show that 50% reductions of any one MCM is well-tolerated but decreases of ∼2/3 are not, supports the idea of a threshold effect, and suggests that the threshold lies somewhere between 1/3 and 1/2 of normal MCM levels (at least in the cases of MCM2, MCM6 and MCM7).

These results also emphasize the importance of relevant physiological models, both in general and with respect to the MCMs. RNAi knockdown of MCM3 in human cells to ∼3% normal levels was still compatible with normal short-term proliferation, although the cells had GIN and high sensitivity to replication stress [16]. It is doubtful such a drastic situation would be recapitulated *in vivo* (it would likely result in embryonic lethality as in *Mcm3^Gt/Gt^* mice). Nevertheless, it is noteworthy in that study that MCM3 depletion was better tolerated than knockdowns of any other member of the replicative helicase.

The finding that reductions in MCM3 rescued MCM2/4/6 depletion phenotypes lends insight into dynamics and regulation of mammalian DNA replication. In budding yeast, MCMs shuttle between the nucleus and cytoplasm during the cell cycle. MCM2-7 multimers must be assembled in the cytoplasm before being imported into the nucleus during G1 phase [4]. The MCM2-7 importation is dependent upon synergistic nuclear localization signals (NLS) on Mcm2 and Mcm3 [22]. In order to prevent over-replication of the genome, MCMs are exported from the nucleus during S, G2 and M [4]. This export is dependent upon Mcm3, which has a nuclear export signal (NES) that is recognized by Cdc28 to promote MCM2-7 export in a Crm1-dependent manner [22].

In contrast to budding yeast, MCMs that have been studied (MCM2/3/7) are primarily nuclear-localized throughout the cell cycle in metazoans and in fission yeast [4]. Upon dissociation from chromatin during S phase, MCM2-7 complexes are reported to remain in the nucleus but are sequestered via attachement to the nuclear envelope or other nuclear structures [24], [33]–[35]. Interestingly, *mcm* mutations in fission yeast that disrupt intact MCM2-7 heterohexamers triggers active redistribution of MCMs to the cytoplasm [36]. Additionally, re-distribution of MCMs between the cytoplasmic and nuclear compartments has been observed in hormonally-treated mouse uterine cells [25].

Our observations support the idea that intracellular re-distribution of MCMs also occurs in mammals, and that it is an important regulatory process. Staining of MCM2 in intact nuclei of normal NIH 3T3 fibroblasts and MEFs show a steady decline (but not elimination) as S phase progresses. Furthermore, it appears that the process of nuclear MCM2 elimination during S phase is regulated, since in situations of decreased MCMs (as in the *Mcm4^Chaos3/Chaos3^* mutant), there is decreased loss of nuclear MCM2 during S phase.

Three lines of experimentation implicate MCM3 as playing a key role in regulating intracellular MCM localization: 1) Rescue of reduced-MCM phenotypes by genetic reduction of MCM3; 2) Increased S-phase nuclear MCM2 by *Mcm3* hemizygosity in MCM-depleted cells (Figure 7D); and increased chromatin-bound MCM2/4 by *Mcm3* hemizygosity in MCM-depleted cells. Our data suggests that MCM3 acts as a negative regulator that prevents re-assembly or reloading of MCM complexes as they dissociate from DNA during replication. As described earlier, mouse and human MCM3 have predicted NESs in similar positions of their primary amino acid sequences as do the yeast genes. Thus, one explanation for these phenomena is that decreased MCM3 suppresses MCM2-7 nuclear export, which occurs normally and which may be accentuated by the *Chaos3* mutation in a fashion analogous to *mcm* mutant fission yeast discussed above [36]. This would effectively increase the amounts of MCMs available for replication licensing. More work is required to determine if the rescue mechanism is indeed related to a decrease in MCMs export, as opposed to direct or indirect involvement in other events such as increased nuclear import or enhanced chromatin loading.

With respect to the early lymphoma susceptibility phenotype in *Mcm4^Chaos3/Chaos3^ Mcm2^Gt/+^* mice, it is unclear whether the type of tumor is dictated primarily by the particular Mcm depletion (in this case MCM2, thus resembling *Mcm2^IRES-CreERT2/IRES-CreERT2^* animals), the genetic background, or the age of particular cancer onset (if animals die of thymic lymphoma at an early age, they will be unable to manifest later-arising mammary tumors). The compound mutant mice used for the aging aspects of this study were bred to at least the N3 generation in strain C3H. *Mcm4^Chaos3/Chaos3^* mice congenic in this background are predisposed exclusively to mammary tumors, whereas lymphomas were observed in mutants of mixed background [17]. Presently, we favor the idea that genetic background and age of tumor type onset are primary determinants of the cancers that arise in the mice we have studied thus far. Genetic background has also been reported to influence tumor latency in MCM2-deficient mice [21].

The MCM2-7 pan-reduction in Chaos3 cells is consistent with other studies involving mutation or knockdown of a single MCM in mammalian cells [16], [20], [29], [37]. In these examples of parallel MCM decreases, the general assumption is that there is hexamer destabilization or impaired MCM chromatin loading followed by degradation of monomers. However, we found that the protein decreases are related to decreased mRNA levels. These large (∼40%) decreases do not appear to be attributable to transcriptional alterations from cell cycle disruptions (these cells have a small elevation in the G2/M population), but rather occur at the post-transcriptional level (unpublished observations). Since we also found that MEFs carrying only 1 functional *Mcm2* allele caused ∼20% decreases of *Mcm3-7* mRNAs, it is possible that mRNA downregulation drove MCM reductions in these other model systems. However, the mechanism for coordinated mRNA regulation, and what triggers it, is a mystery that we are currently investigating.

Our data contribute to a growing body of data that replication stress, which can occur via perturbations of the DNA replication machinery, plays a significant role in driving cancer [38]–[41]. While the *Mcm4^Chaos3^* mutation is an unique case, the deleterious consequences of MCM reductions suggest that genetically-based variability in DNA replication factors can have physiological consequences. Such variability in functions or levels may be caused by Mendelian mutations or multigenic allele interactions. Mutations affecting transcriptional activity of one or more Mcms, which might occur in non-coding *cis*-linked sequences or unlinked transcription factors, could have such effects. This has implications for cancer genome resequencing projects, whereby such mutations would not be obviously associated with MCM expression. The allelic collection we generated, when used alone or in combination with each other or *Mcm4^Chaos3/Chaos3^* mice, allow the generation of mouse models with a graded range of MCM levels. These should be valuable for investigations into the impact of replication stress on animal development, cancer formation, and cellular homeostasis.

## Materials and Methods

### MEF culture and proliferation assays

MEFs from 12.5- to 14.5-dpc embryos were cultured in DMEM+10% FBS, 2 mM GlutaMAX, and penicillin-streptomycin (100 units/ml). Assays were conducted on cells at early passages (up to P3). For cell proliferation assays, 5×10^4^ cells were seeded per well of a 6 well plate. They were then cultured and harvested at the indicated time points to perform cell counts.

### iPS induction from MEFs using lentiviral vectors

Doxycycline inducible lentiviral vectors [42] were prepared by co-transecting viral packaging plasmids psPAX2 and and pMD2.G along with vectors encoding rtTA, *Oct4*, *Sox2*, *Klf4*, or *c-Myc* (plasmids were obtained from Addgene.org, serial numbers 12259, 12260, 20323, 20322, 20324, and 20326) into 293T cells using TransIT-Lt1 transfection reagent (Mirus). Viral supernatants were collected at 48 and 72 hours, and concentrated using a 30kd NMWL centrifugal concentrator. MEFs from 13.5d embryos, up to P3, were seeded to gelatin coated tissue plates at a density of 6.75×10^3^ cells/cm^2^ and allowed to attach in standard MEF media for 24 hours before infection with lentiviral vectors. After 24 hours incubation the culture media was changed to KO-DMEM supplemented with 15% KO serum replacement (Gibco), recombinant LIF, 2 µg/mL doxycycline (Sigma), 100 µm MEM non-essential amino acids solution, 2mM GlutaMax, 100 units/mL penicillin and 100 µg/mL streptomycin (Gibco). The induction media was refreshed daily for 13 days until the cells were passaged to 100 mm plates prepared with irradiated feeders. Cells were cultured for an additional 10 days in the induction media in the absence of doxycyline before iPS colony counting, cell counts, and flow cytometry.

For flow cytometric quantification of iPS cells derived from reprogramming of MEFs, ∼1×10^6^ cells were trypsinized for 10 minutes, then washed twice with cold PBS. They were gently but completely resuspended in 1ml of 4% paraformaldehyde in PBS at room temperature for 30 minutes. The fixed cells were pelleted by centrifugation at 500×G for 2 minutes and washed twice with 10 ml TBS-TX (0.1% Triton X-100) buffer. For antibody staining, the cells were blocked with 1ml TBS-TX buffer with 1% BSA for 15 min at room temperature, then stained with primary “stemness” antibodies (monoclonal anti-SSEA1, Millipore; rabbit polyclonal anti-LIN28, Abcam) for 60 min, washed twice, then secondary antibody was applied for 60 minutes. Immunolabeled cells were analyzed by flow cytometry using a 488nm laser. Secondary antibodies were goat anti-mouse IgG-FITC (South Biotech) and goat anti-rabbit IgG-594 (Molecular Probes). Cells were considered to be iPS cells if they were LIN28/SSEA1 positive. Calibration of the flow cytometer and gates were set using untransfected MEFs as negative controls, and v6.4 ES cells as positive controls.

For quantification by colony formation, plates containing the passaged reprogrammed cells were examined microscopically at 20×, and 4 fields were scored and averaged. Colonies were considered as iPS clones based on morphological criteria: well defined border, three-dimensionality, and tight packing of cells.

### Flow cytometric analyses of micronuclei and iPS cells

Micronucleus assays, which include CD71 staining, were performed essentially as described [43].

### Nuclei isolation and immunofluorescence staining

MEFs were plated at 4×10^6^ cells/150 mm culture dish for 60 hr, trypsinized, then resuspended in 1ml PBS. To the suspension was added TX-NE (320 mM sucrose, 7.5 mM MgCl_2_, 10 mM HEPES, 1% Triton X-100, and a protease inhibitor cocktail). The cells were gently vortexed for 10 seconds and incubated on ice for 30 min. Dounce homogenization was unnecessary. Nuclei were then pelleted by centrifugation at 500×G for 2 min and washed twice with 10 ml TX-NE, then resuspended in 1ml TX-NE. Nuclei yield and integrity was monitored microscopically with trypan blue staining. The nuclei were fixed by adding 15ml cold methanol for 60 min on ice. The fixed nuclei were pelleted by centrifugation at 500×G for 2 min, then washed twice with 10 ml TBS-TX (0.1% TX-100). 1×10^6^ nuclei were placed into 1.5ml tubes in 1ml TBS-TX buffer+1% BSA for 15 min at room temperature. The primary antibody (Rabbit anti-mouse MCM2) was added for 60 min, then secondary antibody (FITC goat anti-rabbit) was added for 60 min. Finally, the nuclei were stained with propidium iodide (PI), and RNAse treated (batches optimized empirically) for 30 mins. Immunolabeled nuclei were analyzed by flow cytometry (using a BD FACSCalibur cytometer with CellQuest software), exciting the PI and FITC with a 488nm laser.

### Generation and validation of mouse lines bearing mutant Mcm alleles

ES cell lines containing gene trap insertions in Mcm genes were obtained from Bay Genomics [*Mcm3* (RRR002), *Mcm6* (YHD248), *Mcm7* (YTA285)] or the Sanger Institute [*Mcm2* (ABO178)]. The *Mcm4* line was previously reported [17]. Allele names are abbreviated as, for example, *Mcm3^Gt^* instead of the full name *Mcm3^Gt(RRR002)Byg^*. All of the original ES cells were of strain 129 origin, and the alleles were backcrossed into C3HeB/FeJ for ≥4 generations.

To identify the exact insertion sites of the gene trap vectors, a “primer walking” procedure was used. This involved priming PCR reactions with :1) a fixed vector primer, and 2) one of a series of primers series corresponding to the intron in which the vector presumably integrated. PCR products were then sequenced. Genotyping of gene-trap-bearing mice was performed either by PCR amplification of the neomycin resistance gene within the vector, or by using insertion-specific assays (Table S2).

### Western blot analysis

Cytosolic and chromatin-bound protein was extracted as described [44]. Antibody binding was detected with a Pierce ECL kit. Band were quantified using NIH Image J software. Antibodies- aMCM2: ab31159 (Abcam); aMCM3: 4012 (Cell Signaling); aMCM4: ab4459 (Abcam); aMCM5: NB100-78261 (Novus); aMCM6: NB100-78262 (Novus); aMCM7: ab2360 (Abcam); aBeta-actin: A1978 (Sigma); aTBP: NB500-700 (Novus).

### Quantitative RT-PCR (qPCR)

Total RNA from P1 MEFs was DNAse I treated, then cDNA was synthesized from 1 µg of total RNA using the Invitrogen SuperScript III ReverseTranscriptase kit with the supplied Olige-dT or random-hexamer primers. qPCR reactions were performed in triplicate on 1 ng or 10 ng of cDNA by using the SYBR power green RT-PCR Master kit (Applied Biosystems; 40 cycles at 95°C for 10 s and at 60°C for 1 min), and real-time detection was performed on an ABI PRISM 7300 and analyzed with Geneamp 5700 software. The specificity of the PCR amplification procedures was checked with a heat-dissociation step (from 60°C to 95°C) at the end of the run and by gel electrophoresis. Results were standardized to *β*-actin. The PCR primers are listed in Table S1.

## Supporting Information

Figure S1 **Homozygous lethality of Mcm gene trap alleles.**
(0.06 MB PDF)

Figure S2 **Synthetic lethal interactions of gene trap alleles.**
(0.07 MB PDF)

Figure S3 **Histopathology of** ***Mcm4^Chaos3/Chaos3^ Mcm2^Gt^/+*** **tumors.**
(4.87 MB PDF)

Figure S4 **Rescue of viability by** ***Mcm3*** **hemizygosity.**
(0.06 MB PDF)

Table S1 ***Mcm4^Chaos3/Chaos3^ Mcm2^Gt^/+*** **tumor pathology.**
(0.08 MB PDF)

Table S2 **PCR primers.**
(0.04 MB PDF)
