# Supplementary material for: Incremental Genetic Perturbations to MCM2-7 Expression and Subcellular Distribution Reveal Exquisite Sensitivity of Mice to DNA Replication Stress
Source: PLoS Genet. 2010 Sep 9;6(9):e1001110. doi: 10.1371/journal.pgen.1001110 (PMC2936539; doi:10.1371/journal.pgen.1001110)
Supplement: Figure S1 — Homozygous lethality of Mcm gene trap alleles. (0.06 MB PDF) [file pgen.1001110.s001.pdf]

Supplemental Figure 1. Homozygous lethality of Mcm gene trap alleles

Cross: *Mcm2*$^{GT/+}$ X *Mcm2*$^{GT/+}$

Genotypes	*Mcm2*$^{+/+}$	*Mcm2*$^{GT/+}$	*Mcm2*$^{GT/GT}$
**Expected Ratio**	1/4	1/2	1/4
**Expected #**	16.75	33.5	16.75
**Observed**	18	49	0

Total Animals 67

ChiSquare *6.09853E-06*

Viability by Birth 0.00% *(0 out of 16.75)*

Cross: Mcm3$^{GT/+}$ X Mcm3$^{GT/+}$

Genotypes	*Mcm3*$^{+/+}$	*Mcm3*$^{GT/+}$	*Mcm3*$^{GT/GT}$
**Expected Ratio**	1/4	1/2	1/4
**Expected #**	9.75	19.5	9.75
**Observed**	14	25	0

Total Animals 39

ChiSquare *0.001392126*

Viability by Birth 0.00% *(0 out of 9.75)*

Cross: Mcm6$^{GT/+}$ X Mcm6$^{GT/+}$

Genotypes	*Mcm6*$^{+/+}$	*Mcm6*$^{GT/+}$	*Mcm6*$^{GT/GT}$
**Expected Ratio**	1/4	1/2	1/4
**Expected #**	8.25	16.5	8.25
**Observed**	10	23	0

Total Animals 33

ChiSquare *0.003731634*

Viability by Birth 0.00% *(0 out of 8.25)*

Cross: Mcm7$^{GT/+}$ X Mcm7$^{GT/+}$

Genotypes	*Mcm7*$^{+/+}$	*Mcm7*$^{GT/+}$	*Mcm7*$^{GT/GT}$
**Expected Ratio**	1/4	1/2	1/4
**Expected #**	9.25	18.5	9.25
**Observed**	14	23	0

Total Animals 37

ChiSquare *0.001675084*

Viability by Birth 0.00% *(0 out of 9.25)*
