# Supplementary material for: Incremental Genetic Perturbations to MCM2-7 Expression and Subcellular Distribution Reveal Exquisite Sensitivity of Mice to DNA Replication Stress
Source: PLoS Genet. 2010 Sep 9;6(9):e1001110. doi: 10.1371/journal.pgen.1001110 (PMC2936539; doi:10.1371/journal.pgen.1001110)
Supplement: Figure S2 — Synthetic lethal interactions of gene trap alleles. (0.07 MB PDF) [file pgen.1001110.s002.pdf]

Supplemental Figure 2 - Synthetic lethal Interactions of gene trap alleles

A ***$Mcm4^{C3/C3}$* X *$Mcm4^{C3}$/+ $Mcm2^{Gt}$/+***

**Geno:**	***C3/C3 M2/+***	***C3/C3 +/+***	***C3/+ M2/+***	***C3/+ +/+***
**Exp Ratio**	1/4	1/4	1/4	1/4
**Exp**	42.5	42.5	42.5	42.5
**Obs**	**12**	44	59	55
Total	170			
ChiSquare	*5.2E-07*			

B ***$Mcm4^{C3/C3}$* X *$Mcm4^{C3}$/+ $Mcm3^{Gt}$/+***

**Geno:**	***C3/C3 M2/+***	***C3/C3 +/+***	***C3/+ M2/+***	***C3/+ +/+***
**Exp Ratio**	1/4	1/4	1/4	1/4
**Exp**	28.25	28.25	28.25	28.25
**Obs**	29	29	27	28
Total	113			
ChiSquare	*0.99*			

C ***$Mcm4^{C3/C3}$* X *$Mcm4^{C3/+}$ $Mcm6^{Gt/+}$***

**Geno:**	***C3/C3 M6/+***	***C3/C3 +/+***	***C3/+ M6/+***	***C3/+ +/+***
**Exp Ratio**	1/4	1/4	1/4	1/4
**Exp**	27.3	27.3	27.3	27.3
**Obs.**	**4**	**32**	41	32
Total	109			
Chi Square	*1.0E-06*			

C3/C3* X *C3/C3 $M6^{Gt}$/+

C3/C3 M6/+	***C3/C3 +/+***
1/2	1/2
40.5	40.5
**14**	**67**
81	
FET *3.9E-09*	

D ***$Mcm4^{C3/C3}$* X *$Mcm4^{C3/+}$ $Mcm7^{Gt/+}$***

**Geno:**	***C3/C3 M7/+***	***C3/C3 +/+***	***C3/+ M7/+***	***C3/+ +/+***
**Exp Ratio**	1/4	1/4	1/4	1/4
**Exp**	37.5	37.5	37.5	37.5
**Obs (wean)**	**14 (1)**	44	50	41
Total	88			
ChiSquare	*0.04*			

C3/C3* X *C3/C3 $M7^{Gt}$/+

C3/C3 M7/+	***C3/C3 +/+***
1/2	1/2
82	82
**48(4)**	**112**
164	
FET *2.8E-06*	

Legend: *$Mcm4^{Chaos3}$* is abbreviated as *$Mcm4^{C3}$*. The X^2 *P* value for relates to deviation of all classes *in toto* from the expected. In(C) and (D), the *P* values are derived from the Fisher's Exact Test (FET) in comparing the *$Mcm4^{C3/C3}$ $Mcm\#^{Gt}$*/+ vs. the *$Mcm4^{C3/C3}$* classes.
