# Supplementary figures and images for: Incremental Genetic Perturbations to MCM2-7 Expression and Subcellular Distribution Reveal Exquisite Sensitivity of Mice to DNA Replication Stress
Source: PLoS Genet. 2010 Sep 9;6(9):e1001110. doi: 10.1371/journal.pgen.1001110 (PMC2936539; doi:10.1371/journal.pgen.1001110)

Supplemental Figure 3 - Histopathology of *Mcm4*<sup>Chaos3/Chaos3</sup> *Mcm2*<sup>Gt/+</sup> tumors

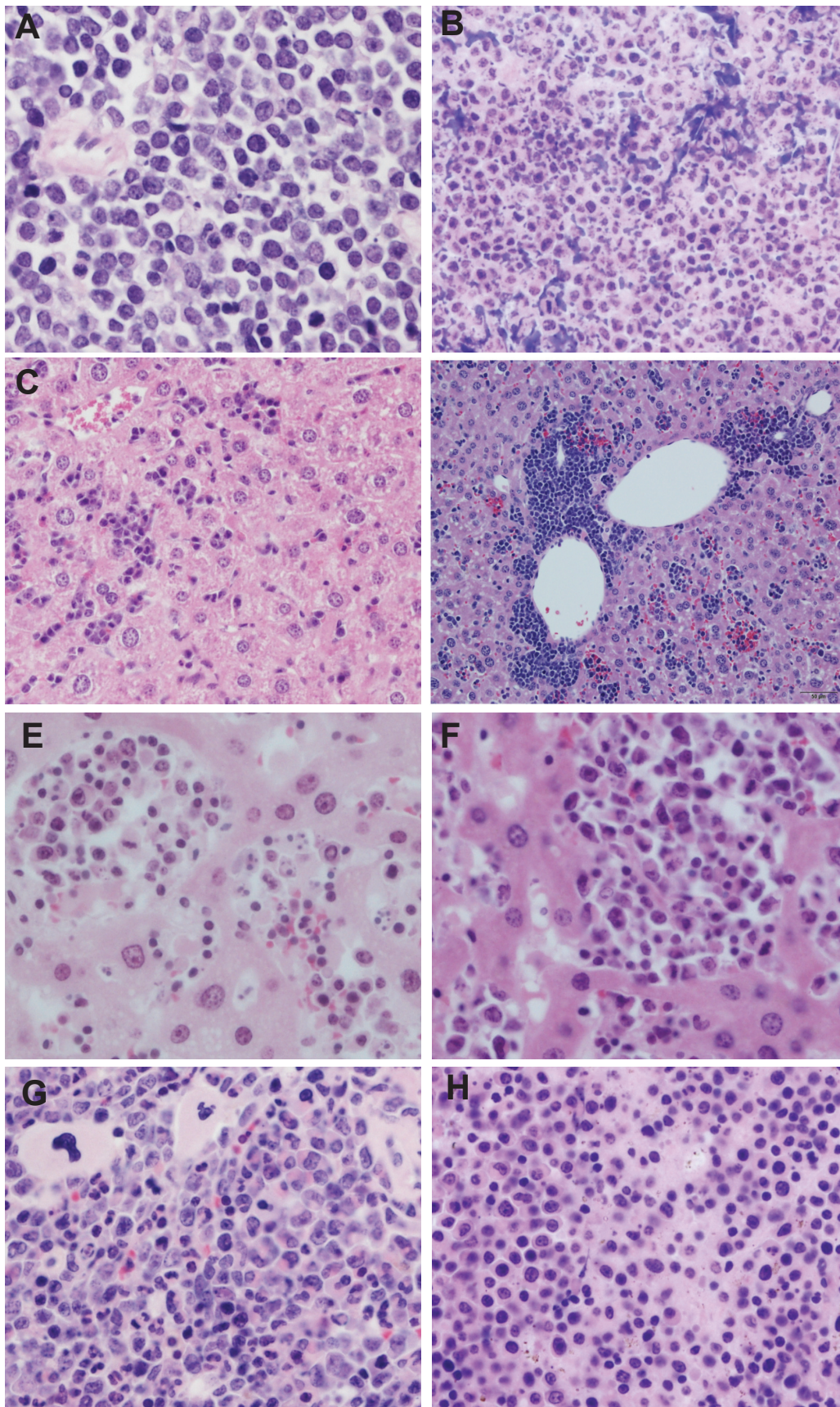

Supplement: Figure S3 — Histopathology of Mcm4Chaos3/Chaos3 Mcm2Gt/+ tumors. (4.87 MB PDF) [file pgen.1001110.s003.pdf]
