# Supplementary material for: Incremental Genetic Perturbations to MCM2-7 Expression and Subcellular Distribution Reveal Exquisite Sensitivity of Mice to DNA Replication Stress
Source: PLoS Genet. 2010 Sep 9;6(9):e1001110. doi: 10.1371/journal.pgen.1001110 (PMC2936539; doi:10.1371/journal.pgen.1001110)
Supplement: Figure S4 — Rescue of viability by Mcm3 hemizygosity. (0.06 MB PDF) [file pgen.1001110.s004.pdf]

Supplemental Figure 4 -Rescue of viability by *Mcm3* hemizygosity

Cross: *$Mcm4^{C3/C3}$* X *$Mcm4^{C3/C3}$ $Mcm2^{Gt/+}$ $Mcm3^{Gt/+}$*

**Genotype:**	***$M4^{C3/C3}$***	***$M4^{C3/C3}$ $M3^{Gt/+}$***	***$M4^{C3/C3}$ $M2^{Gt/+}$***	***$M4^{C3/C3}$ $M2^{Gt/+}$ $M3^{Gt/+}$***	
Expected Ratio	1/4	1/4	1/4	1/4	
Expected #	52.3	52.3	52.3	52.3	
Observed	66	69	**24**	**50**	FET *P*=7.12E-05
**Total**	**209**	χ2 *P= 2.12558E-05*			

Cross: *$Mcm4^{Gt/+}$* X *$Mcm4^{C3/C3}$ $Mcm3^{Gt/+}$*

**Genotypes:**	***$Mcm4^{C3/+}$***	***$Mcm4^{C3/+}$ $Mcm3^{Gt/+}$***	***$Mcm4^{C3/Gt}$***	***$Mcm4^{C3/Gt}$ $Mcm3^{Gt/+}$***	
Expected Ratio	1/4	1/4	1/4	1/4	
Expected #	61.75	61.75	61.75	61.75	
Observed	99	101	**7**	**40**	FET *P*=7.14E-05
**Total**	**247**	χ2 *P= 2.58374E-22*			

Cross: *$Mcm4^{C3/C3}$* X *$Mcm4^{C3/C3}$ $Mcm3^{Gt/+}$ $Mcm6^{Gt/+}$*

**Genotypes:**	***$Mcm4^{C3/C3}$***	***$Mcm4^{C3/C3}$ $Mcm3^{Gt/+}$***	***$Mcm4^{C3/C3}$ $Mcm6^{Gt/+}$***	***$Mcm4^{C3/C3}$ $Mcm3^{Gt/+}$ $Mcm6^{Gt/+}$***	
Expected Ratio	1/4	1/4	1/4	1/4	
Expected #	27.0	27.0	27.0	27.0	
Observed	35	47	**9**	**17**	FET *P*= 0.15
**Total**	**108**	χ2 *P= 3.39925E-07*			
