# Supplementary material for: Incremental Genetic Perturbations to MCM2-7 Expression and Subcellular Distribution Reveal Exquisite Sensitivity of Mice to DNA Replication Stress
Source: PLoS Genet. 2010 Sep 9;6(9):e1001110. doi: 10.1371/journal.pgen.1001110 (PMC2936539; doi:10.1371/journal.pgen.1001110)
Supplement: Table S1 — Mcm4Chaos3/Chaos3 Mcm2Gt/+ tumor pathology. (0.08 MB PDF) [file pgen.1001110.s005.pdf]

Supplemental Table 1. *Mcm4*$^{Chaos3/Chaos3}$ *Mcm2*^{Gt}/+ Tumor Pathology

Mouse	Sex	Age (mo)	Information	Histo
13693	F	4.7	Chest Tumor. Histology indicates thymic lymphoma, with large round cells. Histo:Rib marrow has large infiltrate of blastic cells but still some megakaryocytes; lymphoma in ribs, heart, spleen, liver. Lung-has increased circulating nucleated cells.	Fig. S3a Chest Tumor
14356	F	3.5	Runty. No significant findings in ribs, heart, skeletal muscle, or lungs. Multifocal emphysematous change/bullae	
14358	F	4.6	Runty. Histology- Suggests leukemia. Diffuse enlargement of the spleen. Hepatic sinusoids expanded and spleen effaced by neoplastic round cells, most of which are mononuclear, consistent with lymphoid cells.	Fig. S3b Spleen
14682	F	3.3	Histology: Suspect Leukemic. Sinusoids expanded and spleen effaced by neoplastic cells. Lymphoid vs. myeloid undetermined.	
14691	F	2.4	Large chest tumor. Histology confirmed lymphoma, probably thymic.	
16038	F	3.8	Animal found dead. Cause of death undetermined.	
17168	F	4.8	Runty. Enlarged Inguinal & especially Brachial lymph nodes. Enlarged spleen, pale liver. Histology Spleen: red pulp is expanded by neoplastic round cells mixed with myeloid and erythroid extramedullary hematopoiesis. Liver: sinusoids multifocally contain aggregates of hematopoietic cells. There are neoplastic cells along the capsular surface and in subcapsular vessels. Lymph node: abundant myeloid precursor cells in the medullary cords--Interpretation: round cell tumor, favor granulocytic leukemia.	Fig. S3c Liver
17172	F	2.3	Chest Tumor; mouse found dead. Possible thymic lymphoma.	
18665	F	4.2	Animal found dead. Cause of death undetermined.	
19886	F	2.0	Enlarged Inguinal & Superficial cervical lymph nodes	
13510	M	3.0	Enlarged lymph nodes. Histology of lungs, spleen, liver and anti-CD3 IHC confirmed T cell leukemic lymphoma. See Fig. 6B. In liver, neoplastic cells surround central veins and expand sinusoids.	Fig. S3d Liver
14362	M	4.7	Chest Tumor. Hepatic sinusoids were expanded by neoplastic round cells. Favor leukemic lymphoma but may be myeloid.	
14661	M	5.1	Chest Tumor. Enlarged Inguinal lymph nodes. Histology indicates thymic lymphoma.	
14663	M	6.8	Animal found dead. Enlarged spleen. Possible enlarged Inguinal lymph nodes. Autolysis provented accurate histological analysis.	
14665	M	5.4	There is a focal aggregate of round cells in the adipose tissue near the heart, but impossible to determine the origin of the cells.	
14668	M	5.8	Histology: Pleomorphic cells expanding hepatic sinusoids, most are mononuclear, suggesting lymphoid. Cervical lymph node and spleen are effaced by neoplastic mononuclear cells, most likely lymphocytes. Bone marrow is highly cellular, but mixed population, focally extends into adjacent skeletal muscle.	Fig S3e Liver
14670	M	3.7	Found dead. Tissue was autolyzed and nondiagnostic. No sign of neoplasia.	
14674	M	4.0	Found dead. Tissue was autolyzed and nondiagnostic. No sign of neoplasia.	
14676	M	3.4	Enlarged superficial and Inguinal Lymph nodes. Histology: Polymorphic round cells effacing architecture of spleen and expanding hepatic sinusoids. Interpretation: lymphoma or myeloproliferative disease.	Fig. S3f Liver
15485	M	3.8	Runty. Very enlarged spleen. Histology: Spleen and bone marrow diffusely effaced by neoplastic round cells. Consistent with lymphoma.	
15486	M	2.7	Large Chest Tumor; likely a thymic lymphoma. Tissue effaced by sheets of neoplastic cells.	
16043	M	3.2	Runty. .Probable lymphoma. Very enlarged spleen (3.5 cm), possible enlarged thymus and pancreatic lymph. Histology: neoplastic round cells effacing thymus, lymph nodes and bone marrow, invading skeletal muscle.	
16299	M	3.7	Enlarged spleen. Histology: monomorphic population of round cells arranged in sheets in lymph node, suggestive of lymphoma.	

16300	M	5.5	Enlarged Spleen. Histology: Spleen: red and white pulp effaced by large, pleomorphic neoplastic round cells (lymphoid vs. myeloid vs. histiocytic) mixed with clusters of extramedullary hematopoiesis. Interpretation: round cell neoplasm, favor granulocytic lymphoma.	Fig S3g Spleen
16304	M	4.9	Very enlarged Spleen (4cm x 1.3 cm); Histology: Spleen diffusely effaced by neoplastic round cells. Probably lymphoma.	Fig S3h
16306	M	5.1	Enlarged spleen (3.8 cm x 1 cm). Enlarged L. Inguinal, Superficial Cervical, & pancreatic lymph nodes, effaced by sheets of neoplastic round cells consistent with lymphoma.	
16309	M	2.8	Animal found dead. Enlarged Inguinal Lymph node and Spleen. Nodes autolyzed and non diagnostic.	
