# Supplementary material for: Incremental Genetic Perturbations to MCM2-7 Expression and Subcellular Distribution Reveal Exquisite Sensitivity of Mice to DNA Replication Stress
Source: PLoS Genet. 2010 Sep 9;6(9):e1001110. doi: 10.1371/journal.pgen.1001110 (PMC2936539; doi:10.1371/journal.pgen.1001110)
Supplement: Table S2 — PCR primers. (0.04 MB PDF) [file pgen.1001110.s006.pdf]

Name	Sequence 5'->3'	Usage
mMcm2 mRNA-F (2Q1)	AGA AGT TCA GCG TCA TGC GGA GTA	mRNA qPCR
mMcm2 mRNA-R (2Q2)	CCC AAA GCG GTT GCG TTG ATA TGT	
mMcm3 mRNA-F (3Q1)	AGG AAG ACT CAT GCC AAG GAT GGA	mRNA qPCR
mMcm3 mRNA-R (3Q2)	TGG GCT CAC TGA GTT CCA CTT TCT	
mMcm4 mRNA-F (4Q1)	ACA GGA ATG AGT GCC ACT TCT CGT	mRNA qPCR
mMcm4 mRNA-R (4Q2)	AAA GCT CGC AGG GCT TCT TCA AAC	
mMcm5 mRNA-F (5Q1)	CTG GAT GCT GCT TTG TCT GGC AAT	mRNA qPCR
mMcm5 mRNA-R (5Q2)	TGT GTT CAG ACA CCT GAG AGC CAA	
mMcm6 mRNA-F (6Q1)	TCA CCA AGT CCT CGT GGA GAA TCA	mRNA qPCR
mMcm6 mRNA-R (6Q2)	TTT AGG CTG AAC CTC GTC ACA GCA	
mMcm7 mRNA-F (7Q1)	CCC TGC CCA ATT TGA ACC TTT GGA	mRNA qPCR
mMcm7 mRNA-R (7Q2)	TCT CCA CAT ATG CTG CGG TGA TGT	
mbeta-actin Frd RT	CAT CCT CTT CCT CCC TGG AGA AGA	mRNA qPCR
mbeta-actin Rev RT	ACA GGA TTC CAT ACC CAA GAA GGA AGG	
IMR13	CTT GGT GGA GAG GCT ATT C	beta-geo genotyping PCR
IMR14	AGG TGA GAT GAC AGG AGA TC	
IMR15	CAA ATG TTG CTT GTC TGG TG	postive control for genotyping PCR
IMR16	GTC AGT CGA GTG CAC AGT TT	
ABO178-R (2G2)	GCA GTA GAG TTC CCA GGA GGA GCC	ABO178 genotyping PCR
ABO178-F (2G1)	CCC TCC TCC TGC AGG TGG AAA GCA C	
ABO178-WR (2G3)	GGT GGT GTA AGG AAC AGA TGG AC	
RR0002-R (3G2)	GGA TGA GGG AGC AGG GCT CGG CAC	RRR002 genotyping PCR
RR0002-F (3G1)	CAC TGT TTA TAT GTG CAC GTG TAC C	
RR0002-WR (3G3)	CTT CTG TCG CTT TCA GAC CAG AAG C	
RRE056WR (4G3)	GAC ACA TAA ATC TTG CCA ATG AGG	RRE056 genotyping PCR
Mcm4 e12L (4G1)	AAA TGT CAG ATG AAG ATG GTT T	
Vector R (4G2)	CCA TAC AGT CCT CTT CAC ATC CAT GC	
Mcm6e9L2 (6G1)	GAC GTT GAA TCC GTT TTA GA	YHD248genotyping PCR
Mcm6e9R (6G3)	GCA CCA ACT AAT GAT GAG TCT T	
YHD248R (6G2)	AGT AGA TCC CGG CGC TCT TAC CAA	
YTA185R (7G2)	GGG AAA GAG GGC TCT GTC CTC CAG	YTA285 genotyping PCR
mMcm7intron1F (7G1)	AAG GGA CTA GGT TAC TGG GCC AGC	
mMcm7intron1R (7G3)	CCT TTC GCC ACC GGG CGC TTA GCC	
Chaos3type831L	CAT TGA TCA GCT CAT CAC CA	Chaos3 mutant genotyping PCR
Chaos3typeR	CAC ATA CCA TTT GCT TGT CAG	
